# The TONK score: a tool for assessing quality in trauma and orthopaedic note-keeping

**DOI:** 10.1051/sicotj/2015029

**Published:** 2015-11-02

**Authors:** Zeeshan Khan, Adele E. Sayers, Mohammad Usman Khattak, Iain Richard Chambers

**Affiliations:** 1 Department of Trauma & Orthopaedics, Rehman Medical Institute Peshawar Pakistan; 2 Northern Lincolnshire & Goole Hospitals NHS Trust, Scunthorpe General Hospital, Cliff Gardens Scunthorpe DN15 7BH UK

**Keywords:** Quality, Note-keeping, Audit, Legibility

## Abstract

*Introduction*: Medical case notes are the only lasting interpretation of a patient-physician interaction and are important for good quality patient care. Accurate, legible and contemporaneous note-keeping is important however it can be substandard. This can lead to errors in handover of patients and to medicolegal vulnerability. We present a comprehensive auditing tool for Trauma & Orthopaedics medical case notes and our experience in using it over the last 12 months.

*Patients and Methods*: The TONK score was developed from a pre-existing system with some additions for Trauma & Orthopaedic case notes, with the incorporation of a legibility scoring system. An initial audit was carried out evaluating the case notes for each team against the TONK score. In order to evaluate the reproducibility of this score, we employed the Cohen’s Kappa coefficient and noted substantial agreement. The individual team scores were analysed and the audit cycle completed four months later with the provision of feedback.

*Results*: Our first audit revealed a mean of 81 with a range from 70 to 90. Subsequent audits over the next two quarters revealed mean scores in excess of 90. Significant improvement has been noted in all areas of documentation and it has been decided to conduct this audit every six months in our department.

*Conclusions*: The TONK score is an easy, quick and reproducible tool, which aims to eliminate the weaknesses in Trauma & Orthopaedic medical note-keeping. It emphasises the medicolegal importance of accurate medical note-keeping to doctors at all levels of training.

## Introduction

The importance of accurate, legible and up-to-date medical note-keeping cannot be denied for the continuity of good quality patient care. Its importance is further enhanced by its role in audit, research and in medical negligence claims. The Royal College of Surgeons of England published their guidelines for clinicians on medical records and note-keeping in 1990 and revised them in 1994. Its importance for patient care, audits and professional development is stressed [1]. The General Medical Council (GMC), UK, also stresses the importance of good medical note-keeping in its Guide to Good Medical Practice [2].

Much has been said and published not only about the importance of comprehensive medical notes but also about their legibility. Many medicolegal cases have been successful for the claimants due to deficiencies and illegibility of the medical notes [3, 4].

The majority of note-keeping is done by junior doctors, who only appreciate its importance later in their career. This is why this practice should be evaluated regularly with quick, easy and reliable methods. With the introduction of the European Working Times Directive (EWTD) and shift patterns, the importance of this issue is further highlighted and has been evaluated [5]. The problem of poor note-keeping can be further compounded by scarcity of junior doctors and locum doctors on shifts.

**Figure 1.**
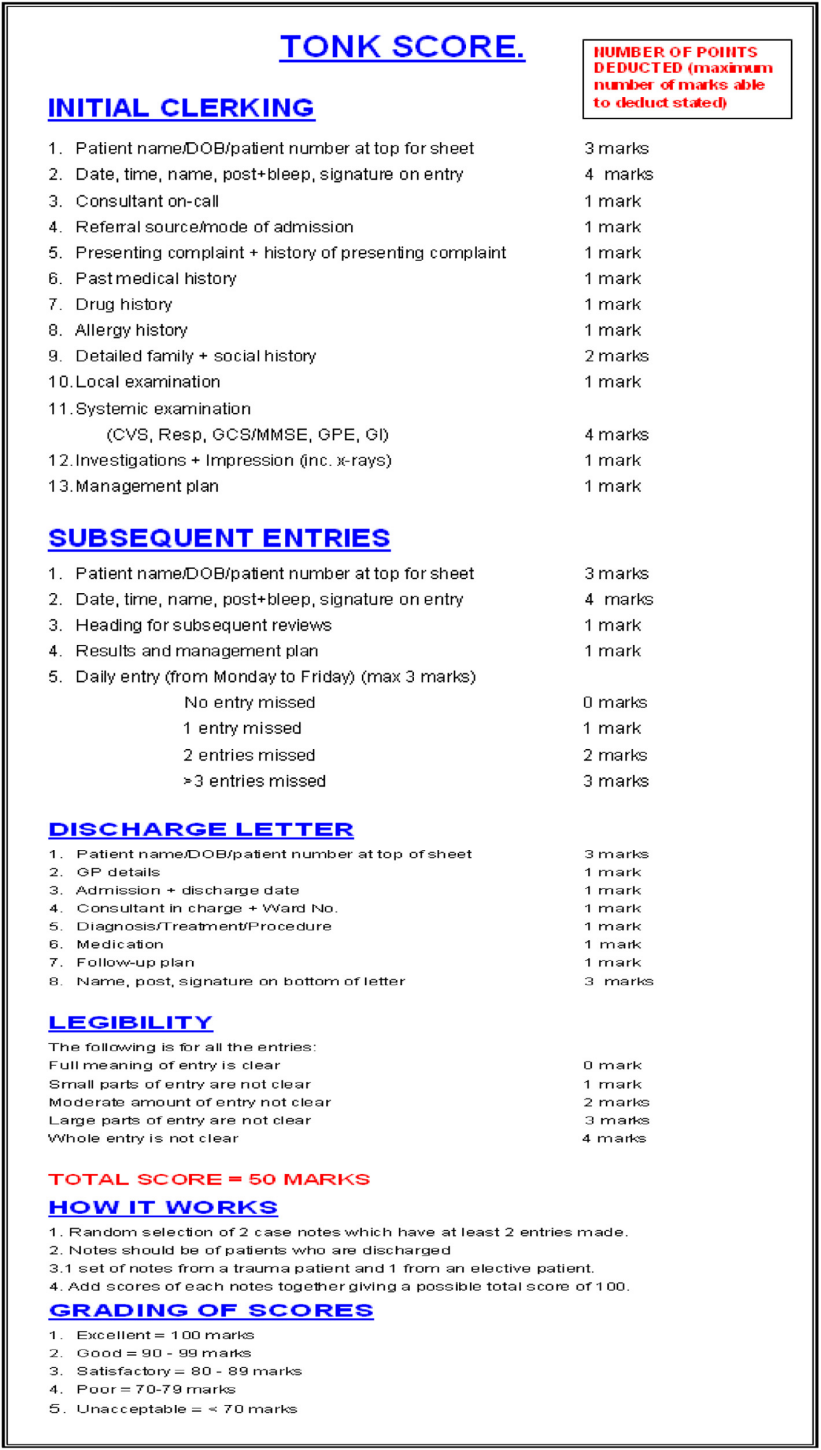
The TONK score form with the scoring system explained.

We propose the TONK score, which comprehensively covers all the important aspects that should be present in the medical notes of a Trauma & Orthopaedics patient. This system has been developed from a pre-existing generic scoring system with certain modifications which are relevant and important to the specialty of Trauma and Orthopaedics [6]. An objective legibility score has also been incorporated, which was lacking in the previous system (Figure 1). This system has been checked for inter- and intra-observer variability by using the Cohen’s Kappa coefficient, and was found to have significant agreement [7].

To our knowledge, this is the only evaluation tool for Trauma & Orthopaedic medical note-keeping and the only scoring system, which has been statistically proven for its reproducibility. We propose that the adoption of the TONK score will not only improve the quality of Trauma & Orthopaedic medical note-keeping but also significantly help in reducing any weaknesses for legal issues.

## Methodology

The TONK was generated by using guidelines published by the Royal College of Surgeons of England and the General Medical Council, UK [1, 2]. It is a four part auditing tool that assesses the quality of initial clerking, all subsequent entries made, the discharge letter and the legibility of entries made during an admission. Various aspects of medical clerking were kept under consideration whilst devising this score and particular attention was also paid to Trauma & Orthopaedic specific information. Marks were assigned in the order of importance to every detail.

To audit the quality of a team’s medical note-keeping, two sets of notes for discharged patients from a designated time period are selected randomly, one being from a trauma patient and the other from an elective patient. A total of 50 points are assigned to each set of notes, with a possible overall score of 100 points per team. In order to make the tool easier to use, scoring is based on a system where points are deducted for each aspect within the entry that is missed (Figure 1). A maximum of four marks only can be deducted if one missed out on all the mentioned details in part 2 of the initial clerking or subsequent entries section. In the end, scores are graded as less than 70% being unacceptable to 100% being excellent.

We introduced this scoring system into our unit when we conducted the first phase of our audit. Two independent scorers, unaware of the trial, were asked to mark the medical notes. They were later asked to mark the notes again after two weeks. These scores were checked for inter- and intra-observer variability employing the Cohen’s Kappa coefficient and measured at 0.71 (substantial agreement) [7]. After evaluating and confirming the validity of this score, we conducted the first phase of the audit and later completed the audit loop after four months. This audit is now run regularly on a quarterly basis in our unit.

## Results

**Figure 2.**
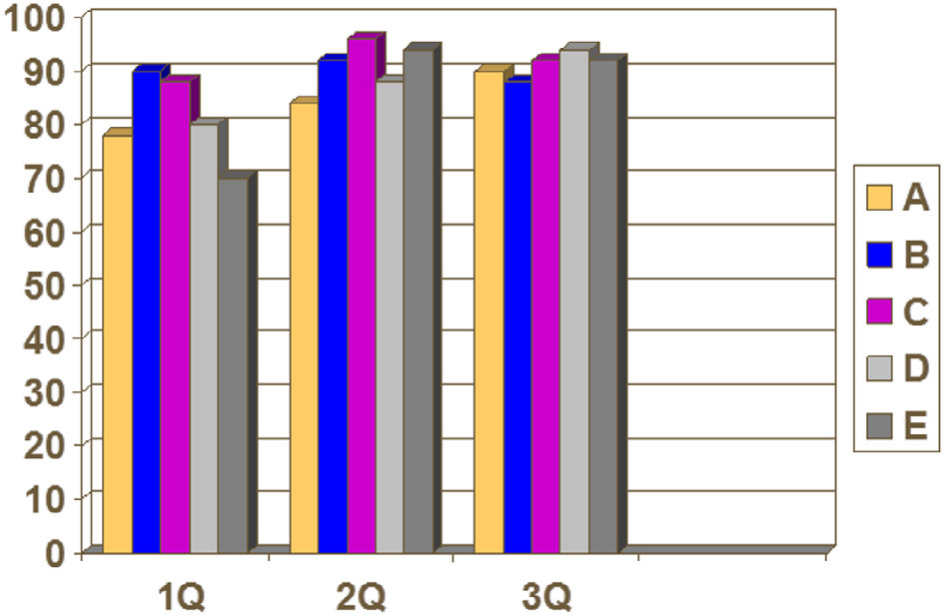
Results of all five teams during the three quarters.

For the purpose of this study, our unit was divided into five teams and each team’s medical notes were marked by independent observers in order to eradicate bias. The study period was over three quarters. The initial phase revealed scores ranging from 70 to 90, with a mean score of 81.2. In the next quarter the mean score improved to 90.8 with the minimum score being 84. In the last quarter the mean score improved to 91.2 with a range of 88–94 (Figure 2).

The areas of weakness mainly noted were in the initial clerking and subsequent entries section, but they all improved in the later phases. The main areas of concern were the timing of the entry, source of admission, name of the consultant on call and details of the person making the entry. Significant improvements were also noted in the documentation of social history and legibility of documentation.

## Discussion

Medical records are an integral part of effective patient care. They are used not only for primary clinical purposes but also for secondary purposes including reporting the activity of hospital services, monitoring performance of hospitals and for research. They remain the most important focus of any patient complaint or litigation.

Poor standards of clinical documentation plague all specialities. Maintaining good standards of clinical documentation remains a problem in the health service despite continued and consistent advice from protection organisations and professional bodies over many years [8]. The Medical Protection Society reports many cases which have been successfully won by the claimant because the medical notes were inadequate to successfully defend the case [3].

Incomplete documentation and illegible handwriting with confusing abbreviations in the notes are a major problem. Studies have found that the handwriting of doctors was significantly less legible than other healthcare professionals and time constraints was reported to be one of the causes of poor legibility [9, 10].

We found there was a need for an auditing tool to assess the quality of medical note-keeping in Trauma & Orthopaedics, which should be quick, easy, reliable and reproducible. The TONK score was hence constructed after reviewing the Royal College of Surgeons of England and the GMC, UK guidelines’. This system, to the author’s knowledge, is the only scoring system for medical note-keeping which has been validated and checked for its reproducibility. This audit has now become a regular part of our unit’s audit meetings and has shown significant improvement in the quality of note-keeping and created a healthy competitive environment between the firms. Liyanage et al. also reported improvements in their notes keeping in ophthalmic casualty following regular audits [11].

In some units, there will be dictations which will be typed, pasted or stapled to medical records. Although, they will be legible, they should be checked for errors, corrected and signed by the doctor who dictated them. There is also a risk of losing these, if not properly filed in the records. Any errors in medical notes should be scored out with a single line with additions separately dated, timed and signed with the details of the person. Some trusts are already using name stamps with GMC numbers for doctors to help improve documentation.

The CRABEL score has since been validated as an auditing tool by Dhariwal and Gibbons [12]. By using the CRABEL score as a backbone to a new scoring system, the TONK score was hence constructed, after reviewing the Royal College of Surgeons of England and GMC, UK guidelines’. This system, to the author’s knowledge, is the only scoring system for Trauma & Orthopaedics medical note-keeping which has been validated and checked for its reproducibility. This audit has now become a regular part of our unit’s audit meetings and has shown significant improvement in the quality of note-keeping and also created a healthy competitive environment between the firms.

Tuffaha et al. produced a similar scoring system based on the CRABEL score [13]. This scoring system (STAR score) also takes into account the quality of the consenting process and operation note. Within our NHS Trust, the consent and operation notes follow standardised proformas, so we did not include these aspects of the medical notes within our scoring system. The STAR scoring system was validated by assessing its ability to consistently produce the same score when tested on a set of notes. This differs from that of the TONK score, as the Cohen’s Kappa coefficient was used to test inter-rater variability. Both are robust statistical methods used to test the validity and reproducibility of auditing tools.

TONK score also offers itself as a template for complete and detailed notes keeping along with being an auditing tool. Proformas can also reduce the amount of handwriting with the introduction of checkboxes, which can make note-keeping more robust. We propose the regular use of TONK score in every Trauma & Orthopaedic unit across the country will greatly enhance the quality of note-keeping.

## Conclusion

Good medical record keeping is essential for medicolegal reasons. Constant vigilance is required to maintain its highest standards. TONK score is an easy, quick and reliable tool to assess the quality of both contents and legibility of medical notes in Trauma & Orthopaedics. Our experience with it has resulted in significant improvement in the quality of note-keeping and identified areas requiring improvement. Its regular use in audit meetings will serve as a source of feedback for the junior doctors which would help them in their training.

## Conflict of interest

The authors declare that they have no conflict of interest.
